# Identification of main malaria vectors and their insecticide resistance profile in internally displaced and indigenous communities in Eastern Democratic Republic of the Congo (DRC)

**DOI:** 10.1186/s12936-020-03497-x

**Published:** 2020-11-23

**Authors:** Jeanine A. C. M. Loonen, Dominic B. Dery, Bertin Z. Musaka, Janvier B. Bandibabone, Teun Bousema, Marit van Lenthe, Biserka Pop-Stefanija, Jean-François Fesselet, Constantianus J. M. Koenraadt

**Affiliations:** 1 Médecins Sans Frontières (MSF), Amsterdam, The Netherlands; 2 Département de Biologie, Centre de Recherche en Sciences Naturelles (CRSN/Lwiro), Bukavu, South Kivu Democratic Republic of the Congo; 3 Department of Medical Microbiology, Radboud Institute for Health Sciences, Radboud University Medical Center, Nijmegen, The Netherlands; 4 Department of Infectious and Tropical Diseases, London School of Hygiene and Tropical Medicine, London, UK; 5 Laboratory of Entomology, Wageningen University and Research, Wageningen, The Netherlands

**Keywords:** *Anopheles gambiae*, *Anopheles funestus*, Insecticide resistance, Democratic Republic of the Congo (DRC), *Plasmodium falciparum*, Pyrethroids, Malaria, Internally displaced people

## Abstract

**Background:**

Malaria remains a major public health concern in the Democratic Republic of the Congo (DRC) and its control is affected by recurrent conflicts. Médecins Sans Frontières (MSF) initiated several studies to better understand the unprecedented incidence of malaria to effectively target and implement interventions in emergency settings. The current study evaluated the main vector species involved in malaria transmission and their resistance to insecticides, with the aim to propose the most effective tools and strategies for control of local malaria vectors.

**Methods:**

This study was performed in 52 households in Shamwana (Katanga, 2014), 168 households in Baraka (South Kivu, 2015) and 269 households in Kashuga (North Kivu, 2017). *Anopheles* vectors were collected and subjected to standardized Word Health Organization (WHO) and Center for Disease Control (CDC) insecticide susceptibility bioassays. Mosquito species determination was done using PCR and *Plasmodium falciparum* infection in mosquitoes was assessed by ELISA targeting circumsporozoite protein.

**Results:**

Of 3517 *Anopheles* spp. mosquitoes collected, *Anopheles gambiae *sensu lato (*s.l*.) (29.6%) and *Anopheles funestus* (69.1%) were the main malaria vectors*. Plasmodium falciparum* infection rates for *An. gambiae* s.l. were 1.0, 2.1 and 13.9% for Shamwana, Baraka and Kashuga, respectively. *Anopheles funestus* showed positivity rates of 1.6% in Shamwana and 4.4% in Baraka. No *An. funestus* were collected in Kashuga. Insecticide susceptibility tests showed resistance development towards pyrethroids in all locations. Exposure to bendiocarb, malathion and pirimiphos-methyl still resulted in high mosquito mortality.

**Conclusions:**

This is one of only few studies from these conflict areas in DRC to report insecticide resistance in local malaria vectors. The data suggest that current malaria prevention methods in these populations are only partially effective, and require additional tools and strategies. Importantly, the results triggered MSF to consider the selection of a new insecticide for indoor residual spraying (IRS) and a new long-lasting insecticide-treated net (LLIN). The reinforcement of correct usage of LLINs and the introduction of targeted larviciding were also included as additional vector control tools as a result of the studies.

## Background

Malaria is a major public health threat in the Democratic Republic of the Congo (DRC) and places the country among the highest malaria-endemic countries in Africa [1]. The prevalence of malaria has been high throughout the country, with an estimated 25 M (15.7–38.5 M) confirmed malaria cases and 46.8 K (36.2–57.3 K) estimated deaths in 2017 [2, 3]. Effective malaria control is affected by conflict situations the country has experienced over the years, especially in the eastern part of the country [2, 4].

The malaria vectors in DRC include *Anopheles arabiensis, Anopheles coluzzii, Anopheles gambiae *sensu stricto (s.s.),* Anopheles melas, Anopheles funestus* s.s.*, Anopheles rivulorum, Anopheles leesoni, Anopheles confusus, Anopheles nili* and *Anopheles moucheti* [5]. *Plasmodium falciparum* accounts for the majority of malaria cases in DRC [6]. Long-lasting insecticide-treated nets (LLINs) are one of the key vector control measures in the country. However, LLINs and other vector control measures are hampered by the development of insecticide resistance. Resistance to insecticides encompasses physiological, biochemical, molecular and behavioural mechanisms [7, 8]. One of the molecular resistance mechanism against pyrethroids and dichloro-diphenyl-trichloroethane (DDT) are the knockdown resistance (*kdr*) mutations. Different substitutions in the amino acid sequence in the voltage gated sodium channel (*Vgsc*) can disrupt the activity of these insecticides [9]. Two well-known *Vgsc* point mutations are L1014F, which was first detected in West Africa, and L1014S, which was first detected in East Africa [10–12]. Recent findings confirm that L1014F and L1014S are not geographically limited and also occur in DRC [13–16].

The planning and implementation of a vector control programme requires among other things information on the composition and abundance of the vector species, the proportion of infected mosquitoes and their susceptibility to insecticides [17]. Due to the conflict situation in DRC, relatively limited information on the malaria vectors and their susceptibility to insecticides is available in comparison with other African countries.

According to the United Nations refugee agency (UNHCR) an estimated 37,000 people a day are forced to flee their homes because of conflict and persecution worldwide. In 2018, 70.8 million people were forcibly displaced at a global scale, of which 41.3 million were internally displaced people (IDP) [18]. DRC alone has over 3 million IDPs, living in poor conditions [19]. It is shown that population displacement can have serious implications for malaria transmission, and malaria prevalence is often higher in IDP camps compared to surrounding villages [20]. Unfortunately, little is known about malaria vectors and their resistance status in these areas, as well as about the opportunities for vector control. As such, the current study was carried out in three different provinces in eastern DRC where Médecins Sans Frontières-Operational Centre Amsterdam (MSF-OCA) operates. All three provinces are characterized by ongoing conflicts that has forced people to flee their homes, resulting in a higher risk of disease outbreaks, poor nutrition status and higher exposure to communicable diseases due to poor housing, or even absence of housing. The collapse in basic health care services and access makes the situation more precarious. MSF has been working in the provinces of North Kivu and South Kivu since the early 1990s and in Katanga Province from 2003 until 2016. In North and South Kivu MSF supports primary and secondary health care in the Baraka and Mweso hospital, in several health centres in the area and via mobile clinics. At the time of the studies MSF was using Fendona^®^ (active ingredient: α-cypermethrin) for IRS, and distributed different brands of LLINs, mainly PermaNet^®^ 2.0 (deltamethrin), Olyset^®^ (permethrin) and Duranet^®^ (α-cypermethrin). These LLINs were distributed in a targeted way via antenatal care programmes and to patients younger than 5 years old for out and in-patient department. In Shamwana and Baraka, IRS was carried out twice a year in May and November in the MSF supported health structures and compounds. In Kashuga, besides bi-annual IRS in the MSF supported structures, also the entire IDP and indigenous community received IRS once a year in November.

Despite the various interventions (LLIN distributions, IRS, prompt effective treatment with anti-malarials) rolled-out by MSF in its operational areas, malaria transmission remains a public health challenge. In 2012, MSF initiated malaria research studies to understand the persisting high incidence of malaria with indications of increased malaria caseload. These studies included adherence to and efficacy of treatment, coverage and use of bed nets and vector susceptibility to insecticides [21, 22]. The main objective of these studies was to maximise and better target available interventions. The current study was designed to investigate malaria transmission dynamics under emergency settings, with a focus on key entomological indicators used to characterize the risks of malaria.

## Methods

### Study area

**Fig. 1:**
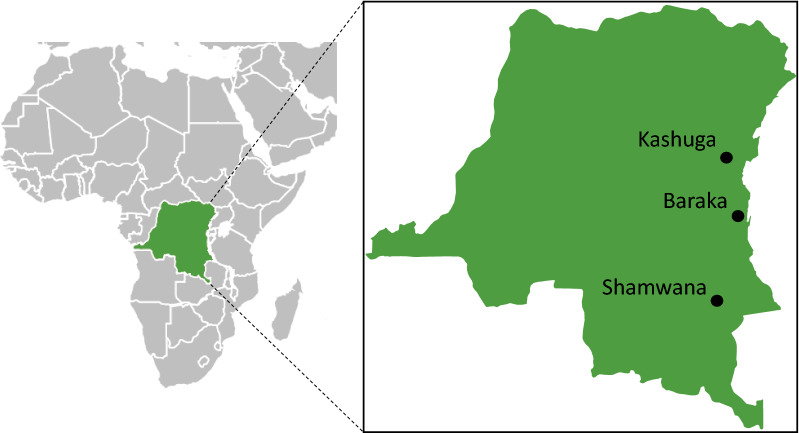
Map of the Democratic Republic of the Congo indicating the three study sites

Mosquito collections were performed at three different locations in the eastern provinces of DRC (Fig. 1). These collections were performed in Shamwana (S08°09.329′, E027°59.365′), Katanga province from March to April 2014, and in Baraka (S04°06.507′, E029°05.728′), South Kivu from June to November 2015. In Kashuga (S01°03.323′, E028°59.255′), North Kivu, collections started in November 2015 but due to insecurity it was interrupted and repeated from January to May 2017. The number of samples collected in Kashuga in 2015 was limited and therefore they were excluded in the analysis. In Shamwana and Baraka indigenous people and IDPs live together. In Kashuga, three IDP camps are present at the borders of the town, while indigenous people mostly live in the centre of the village.

### Study design and sampling procedures

Centers for Disease Control (CDC) Miniature Light Traps (John W. Hock Company, model 512) were used to collect mosquitoes during night time. They were installed inside the bedrooms of houses of both indigenous and displaced people. Houses were randomly selected at the three locations. In Shamwana, 52 houses were selected (out of around 7200 households in total), in Baraka 168 houses (out of around 13,000 households in total) and in Kashuga 269 houses (out of around 4500 households in total) were selected. The houses were geo-referenced with a hand-held GPS device. Traps were placed approximately 1.2 m above the floor at the foot-end of the persons who slept in the room and who consented to receive traps. Traps were switched on by community health workers from around 6:00 pm and removed by 6:00 am the next day. All participants slept under their own bed net. In case they did not own a net, the study team provided a bed net. In the field, collected mosquitoes were identified to species level using the keys of Gillies and de Meillon, and abdominal state of the mosquitoes was recorded [23, 24]. Mosquitoes were put into 2 mL micro-centrifuge tubes of which the lids were pierced with a needle. A maximum of ten mosquitoes of the same species and abdominal state were placed in the same tube and placed into zip-lock plastic bags containing desiccants. The samples were shipped to the Laboratory of Entomology, Wageningen University and Research, the Netherlands, for further molecular analyses.

### Molecular species identification

The *Anopheles* mosquitoes collected with the CDC light traps were individually ground with an automated pestle grinder in a solution of 250µL of 1 × PBS (pH 7.4) with 1% Sarcosil (*N*-Lauroylsarcosine sodium salt) and 0.05% Tween 20. *Anopheles gambiae *sensu lato (s.l.) and *An. funestus* s.l. were identified to species level by polymerase chain reaction (PCR) using this solution [25, 26]. In Table 1, the primer sequences used for species identification are shown. Due to the high number of *An. gambiae* s.l. and *An. funestus* s.l*.* collected in Shamwana, a maximum of five mosquitoes per house with the same abdominal state were analysed by PCR. Samples that did not yield a positive signal were tested twice before they were classified as ‘did not amplify’.

**Table 1:** Subset of primers used for species identification

Species complex	Primer	Primer sequence (5′–3′)
*An. gambiae* s.l.	UN	GTG TGC CCC TTC CTC GAT GT
	AR	AAG TGT CCT TCT CCA TCC TA
	GA	CTG GTT TGG TCG GCA CGT TT
*An. funestus* s.l.	UV	TGT GAA CTG CAG GAC ACA T
	FUN	GCA TCG ATG GGT TAA TCA TG
	VAN	TGT CGA CTT GGT AGC CGA AC
	RIV	CAA GCC GTT CGA CCC TGA TT
	PAR	TGC GGT CCC AAG CTA GGT TC
	LEES	TAC ACG GGC GCC ATG TAG TT

### Detection of *Plasmodium falciparum* sporozoites

A circumsporozoite enzyme-linked immunosorbent assay (CS-ELISA) was performed on mosquitoes collected by CDC traps to determine whether mosquitoes were carrying *P. falciparum* sporozoites [27, 28]. Analyses were conducted at Radboud University Medical Centre, Nijmegen, The Netherlands. Pools of five mosquitoes were tested, and when a pool turned positive, all mosquitoes of that pool were tested individually. A pool was considered positive if the value of the pool was higher than the value of the average of the negative controls plus three times their standard deviation. In all assays, positive and negative controls were included, and plates were read with a spectrophotometer at 450 nm.

### Mosquito collection for insecticide susceptibility bioassays

In Shamwana, adult mosquitoes were collected alive by indoor resting collections with a hand held mouth aspirator. The mosquitoes were collected from rooms of inhabitants early in the morning (6:00 am–9:00 am) and were used the same day for insecticide susceptibility bioassays (9:30 am–11:30 am). Both fed and unfed females were used in the Shamwana experiment and therefore the results are discussed with this limitation in mind. Initially, a mixture of *An. funestus* and *An. gambiae *s.l. was tested in the insecticide susceptibility bioassays. In the end, most of the tested mosquitoes were *An. funestus* and, therefore, all subsequent analyses were done for *An. funestus* only. In Baraka and Kashuga, *An. gambiae *s.l. larvae were collected from stagnant water bodies in the area. Larval sampling was done from several larval breeding sites to increase the genetic variability of the mosquitoes tested in the insecticide susceptibility bioassays. Larvae were grown to adult mosquitoes in the field laboratory, and fed on a sugar solution. Only female mosquitoes were used in the bioassays.

### WHO insecticide susceptibility bioassay

Standardized World Health Organization (WHO) susceptibility bioassays were performed with mosquitoes collected in Shamwana (collected as adults), Baraka and Kashuga (collected as larvae). Mosquitoes were exposed to different classes of insecticides. In all three locations, DDT 4% (organochlorine), bendiocarb 0.1% (carbamate), permethrin 0.75% (pyrethroid) and deltamethrin 0.05% (pyrethroid) were tested. In Kashuga, three additional insecticides were evaluated: α-cypermethrin 0.05% (pyrethroid), malathion 5.0% (organophosphate) and pirimiphos-methyl 0.25% (organophosphate), since these impregnated papers were only available in Kashuga. The bioassays were performed using WHO susceptibility test kits and procedures were aligned with WHO Pesticides Evaluation Scheme (WHOPES) guidelines [29]. Wild caught female *An. funestus* in Shamwana and laboratory reared non-blood fed 3–6 day old female *An. gambiae *s.l. in Baraka and Kashuga were randomly selected for the insecticide susceptibility test. It was aimed to test a total of 100 mosquitoes per insecticide in 4 replicates as per WHO guidelines [29]. However, due to differences in the availability of mosquitoes, the number of mosquitoes tested per insecticide ranged from a minimum of 60 mosquitoes to a maximum of 178 mosquitoes. Depending on the number of experimental tubes tested, one or two control tubes with a minimum of 12 mosquitoes were run simultaneously. The bioassays were performed at 24.6 °C (min. 19.2 °C and max. 30 °C) and a relative humidity of 67.7% (min. 51% and max. 84.4%).

### CDC bottle bioassay

A standardized CDC bottle bioassay was performed with different concentrations of α-cypermethrin in Baraka and Kashuga [30]. Since MSF was using Fendona^®^ (active ingredient: α-cypermethrin) for IRS and α-cypermethrin impregnated papers were not available during the fieldwork in Baraka, it was decided to perform a CDC bottle bioassay with α-cypermethrin alone. In Kashuga, both WHO tube bioassay and CDC bottle bioassay were performed with α-cypermethrin. Four different concentrations of α-cypermethrin were selected: 200, 20, 0.32 and 0.01 mg/L. The diagnostic dose and time whereby 100% of the mosquitoes are expected to die is 12.5 mg/L α-cypermethrin for 30 min [30]. Non-blood fed 3–5 day old female mosquitoes were tested. In Baraka, a minimum of three replicates per insecticide concentration with a total of 80 *An. gambiae* s.l. were exposed and in Kashuga, two replicates of each 25 *An. gambiae* s.l. were exposed per insecticide concentration. During all assays, a control was used in which mosquitoes were released into a CDC bottle coated with only 95% ethanol, the same solvent as used to dissolve the α-cypermethrin. The assays were performed at 27.7 °C (min. 25.7 °C and max. 29 °C) and a relative humidity of 64.5% (min. 52.3% and max. 77.4%).

### Data analysis

The Sporozoite rate (SR) was calculated as the proportion of anophelines tested positive by CS ELISA. Entomological inoculation rates (EIR) were calculated for the three different locations. In Baraka and Kashuga, the EIR was also calculated for different areas in the town. Since no human landing catches were performed during this study, the mosquito collections by CDC light traps were used to obtain the number of mosquitoes collected per household. Latter number was divided by the average number of people sleeping in the house to obtain an estimate of the number of mosquitoes per person. The EIR was calculated as the product of the sporozoite rate and the average number of anophelines collected by CDC light traps divided by the average number of people sleeping in the house. This product was multiplied by 30 to obtain an estimate for the monthly EIR.

Replicate insecticide susceptibility assay results and CDC bottle bioassay test results were pooled per location and analysed. Percentages were compared to WHOPES recommended ranges. Mosquitoes from assays with a mortality > 98% were considered susceptible, mortalities between 90–98% suggest resistance and a mortality lower than 90% confirms resistance [29]. Mortalities were corrected by Abbott’s formula when the mortality ranged between 5–10% in the control groups. If control mortality in the CDC bottle bioassay was larger than 10% and for WHO susceptibility bioassays larger than 20%, the test was discarded and repeated.

## Results

### Mosquito species composition

In Shamwana 2202 mosquitoes were collected by CDC light traps in 52 bedrooms across the village. Of these, 2015 were anophelines, mainly *An. funestus* (89.2%; Table 2). In Baraka, CDC light traps were installed in 168 bedrooms, and captured 4950 mosquitoes of which 1178 were anophelines. Slightly more *An. funestus* (53.7%) than *An. gambiae* s.l. (43.5%) were collected. In Kashuga 1692 mosquitoes were collected in 269 bedrooms of which 324 were *An. gambiae* s.l. and none were *An. funestus*.

**Table 2:** Numbers of different *Anopheles* species collected in CDC light traps in Shamwana (n = 52 trapping nights), Baraka (n = 168 trapping nights) and Kashuga (n = 269 trapping nights)

	Shamwana (%)	Baraka (%)	Kashuga (%)
*An. gambiae* s.l.^a^	204 (10.1)	512 (43.5)	324 (100)
*An. funestus* s.l.^a^	1798 (89.2)	633 (53.7)	0
*An. pharoensis*^a^	0	3 (0.3)	0
*An. rufipes*	2 (0.1)	1 (0.08)	0
*An. coustani*^a^	1 (0.05)	3 (0.3)	0
*An. moucheti*^a^	10 (0.5)	6 (0.5)	0
*An. vinckei*	0	2 (0.2)	0
*An. malculipalpis*	0	2 (0.2)	0
*An. marshalli*	0	13 (1.1)	0
*An. nili*^a^	0	1 (0.08)	0
*An. demeilloni*	0	2 (0.2)	0
Total	2015	1178	324

^a^Competent malaria vectors (47)

Further analysis of the samples from CDC traps for sibling species composition showed that majority were *An. gambiae* s.s. and *An. funestus* s.s. (Table 3). Samples were tested twice before they were classified as ‘did not amplify’.

**Table 3:** Sibling species composition of the *An. gambiae* and *An. funestus* complex; a subset of *An. gambiae* s.l. primers was used for the identification*,* see Table 1

Species	Sibling species	Shamwana (%) (n = 485)	Baraka (%) (n = 1145)	Kashuga (%) (n = 324)
*An. funestus* s.l.	*An. funestus* s.s.	285 (58.8)	580 (50.7)	n.a
	Did not amplify	80 (16.5)	53 (4.6)	n.a
*An. gambiae* s.l.	*An. gambiae* s.s.	113 (23.3)	492 (43.0)	299 (92.3)
	*An. arabiensis*	0	3 (0.3)	5 (1.5)
	Did not amplify	7 (1.4)	17 (1.5)	20 (6.2)

*n.a.* not applicable

### *Plasmodium falciparum* sporozoite infection rates and entomological inoculation rates (EIR)

**Fig. 2:**
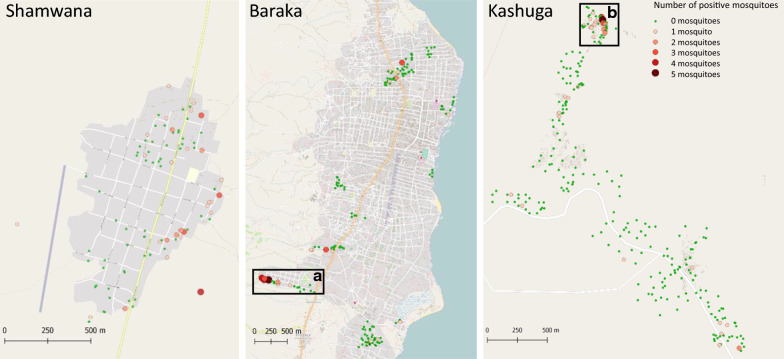
The number of *P. falciparum* sporozoite positive mosquitoes collected per house. **a** Mushimbakye area in Baraka, **b** the north of the Ibuga camp in Kashuga

All *Anopheles* mosquitoes collected by CDC light traps from the three different locations were analysed by CS ELISA to determine whether the mosquitoes were positive for *P. falciparum* sporozoites (Fig. 2). Sporozoite rates in *An. gambiae* s.l. were 1.0% (95% CI 0–2.3%), 2.1% (95% CI 0.9–3.4%) and 13.9% (95% CI 10.12–17.7%) in Shamwana, Baraka and Kashuga, respectively. *Anopheles funestus* were only collected in Shamwana and Baraka and their sporozoite rates were 1.6% (95% CI 1.0–2.2%) in Shamwana and 4.4% (95% CI 2.8–6.0%) in Baraka. In Shamwana, *P. falciparum* was also detected in one *Anopheles coustani*.

Quite strikingly, in both Baraka and Kashuga, the majority of the infected mosquitoes was found in one small area (Fig. 2). In Baraka, 4.2% of all houses (7/168) had more than half (51.3%; 20/39) of all infected mosquitoes. In the north of Kashuga, in one of the IDP camps (Ibuga) 18.6% of all houses sampled (52/269) had two third (66.7%; 30/45) of all infected mosquitoes.

In Shamwana, the EIR was estimated at 4.5 infective bites per person per month, based on 38.8 *Anopheles* mosquitoes per night, a sporozoite rate of 1.3% and 3.4 persons per house (Table 4). To show the heterogeneity of malaria transmission in Baraka, the EIR was calculated for entire Baraka (168 houses), for the area where the highest number of positive mosquitoes were found, called Mushimbakye (20 houses) and for Baraka minus Mushimbakye (148 houses). The EIR for Baraka was 1.2 infective bites per person per month, based on 7.0 *Anopheles* mosquitoes per night, a sporozoite rate of 3.3% and 5.6 persons per house. For the part of Mushimbakye the EIR was considerably higher: 4.2 infective bites per person per month, based on 13.0 *Anopheles* mosquitoes per night, a sporozoite rate of 7.7% and 7.1 persons per house. The EIR for Baraka minus the part of Mushimbakye was 0.7 infective bites per person per month, based on 6.2 *Anopheles* mosquitoes per night, a sporozoite rate of 2.1% and 5.4 persons per house.

**Table 4:** Entomological inoculations rates for the different areas

Location	No. of houses	Sporozoite rate	Average no of *Anopheles*/night	Average no. of people/house	EIR month
Shamwana	52	1.3	38.8	3.4	4.5
Baraka	168	3.3	7	5.6	1.2
Mushimbakye	20	7.7	13	7.1	4.2
Baraka–Mushimbakye	148	2.1	6.2	5.4	0.7
Kashuga	269	13.9	1.2	5.8	0.9
Ibuga	52	13.5	4.3	5.2	3.3
Kashuga–Ibuga	217	14.7	0.5	5.9	0.4

In Kashuga, the EIR was also calculated for different areas: for entire Kashuga (269 houses), for the north of the Ibuga camp alone where the highest number of positive anophelines were found (52 houses) and Kashuga minus the north of the Ibuga camp (217 houses). The EIR for Kashuga was 0.9 infective bites per person per month, based on 1.2 *Anopheles* mosquitoes per night, a sporozoite rate of 13.9% and 5.8 persons per house. For the north of the Ibuga camp the EIR was 3.3 infective bites per person per month, based on 4.3 *Anopheles* mosquitoes per night, a sporozoite rate of 13.5% and 5.2 persons per house. The EIR for Kashuga minus the north of the Ibuga camp was 0.4 infective bites per person per month, based on 0.5 mosquitoes per night, a sporozoite rate of 14.7% and 5.9 persons per house.

### WHO insecticide susceptibility assays

Based on WHO insecticide susceptibility assays, deltamethrin resistance was suspected in Shamwana and Baraka and was confirmed in Kashuga (Table 5). Permethrin resistance was present in all three locations. Bioassays with α-cypermethrin were only done in Kashuga and insecticide resistance was confirmed. In Shamwana mosquitoes were still susceptible to DDT in contrast to Baraka and Kashuga where high resistance levels were shown. Bendiocarb resistance was suspected in Shamwana, however in Baraka and Kashuga local mosquitoes were still susceptible. The organophosphates, pirimiphos-methyl and malathion were only tested in Kashuga, and showed to be effective as the mosquitoes were fully susceptible.

**Table 5:** Mortality of *An. funestus* (Shamwana) and *An. gambiae* (Baraka and Kashuga) exposed to different insecticides

	Shamwana (*An. funestus*)			Baraka (*An. gambiae* s.l.)			Kashuga (*An. gambiae* s.l.)		
	n	Mortality (%)	Resistance status	n	Mortality (%)	Resistance status	n	Mortality (%)	Resistance status
DDT (4%)	78	99	Susceptible	79	9	Resistant	115	21	Resistant
Deltamethrin (0.05%)	81	96	Suspected resistance	60	90	Suspected resistance	178	66	Resistant
Permethrin (0.75%)	85	72	Resistant	76	83	Resistant	119	9	Resistant
α-Cypermethrin (0.05%)	–	–	–	–	–	–	158	50	Resistant
Bendiocarb (0.1%)	81	95	Suspected resistance	80	100	Susceptible	102	99	Susceptible
Pirimiphos-methyl (0.25%)	–	–	–	–	–	–	149	100	Susceptible
Malathion (5%)	–	–	–	–	–	–	119	100	Susceptible

Resistance status is based on WHO criteria (29). A dash (–) indicates that no mosquitoes were evaluated for resistance for that insecticide

### CDC bottle bioassay

**Fig. 3:**
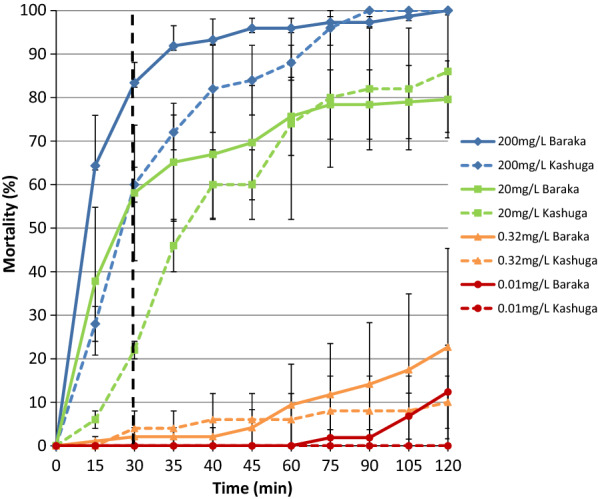
CDC bottle bioassay results performed in Baraka and Kashuga. The black dotted line shows the diagnostic time (30 min) at the diagnostic dose of 12.5 mg/L α-cypermethrin at which 100% mortality is expected. Error bars show the standard error of the mean

In Baraka and Kashuga, *An. gambiae* s.l. were exposed to four different concentrations of α-cypermethrin: 200, 20, 0.32 and 0.01 mg/L (Fig. 3). At the diagnostic dose of 12.5 mg/L α-cypermethrin, more than 98% of the mosquitoes in a susceptible population are supposed to die after 30 min. This exact concentration was not tested in the field but from the data it could be derived that at a concentration of 12.5 mg/L α-cypermethrin the mortality will be less than 58% in Baraka and less than 22% in Kashuga. Furthermore, the highest dose used (200 mg/L) is 16 × the diagnostic dose, and mortality of mosquitoes from both locations at 30 min was under 90%, indicating substantial to high intensity resistance in the local *An. gambiae* s.l. population.

## Discussion

Malaria transmission was high in all three study sites that were inhabited by internally displaced communities. High levels of insecticide resistance to the currently used compounds in LLIN and IRS programmes was observed from the data reported. The main malaria vectors collected during this study were *An. gambiae* s.s. and *An. funestus* s.s., but the dominant species differed per study site (Shamwana: *An. funestus,* Baraka: *An. funestus* and *An. gambiae*, Kashuga: *An. gambiae*)*.* The number of *An. arabiensis* collected was low. Knowing the exact species is important for the implementation of effective vector control interventions, since the vectorial capacities of the sibling species differ [23, 31]. Some samples gave no signal during the PCR analyses, which might be due to poor quality of the DNA extracted or it might result from unidentified species like Stevenson et al*.* demonstrated in their study [32]. It was shown previously that *An. gambiae* s.s. is the predominant vector of the *Gambiae* complex in eastern DRC [33]). This seems in contrast with other areas in East Africa where a switch between the strongly anthropophilic and endophilic *An. gambiae* s.s. to the more opportunistic and exophilic *An. arabiensis* has been observed [34–36]. However, it is debatable whether Eastern DRC should climatically be considered part of East Africa. Mosquito populations will probably be genetically different because of the separation by the big lakes on the borders. The higher presence of *An. gambiae* s.s. compared to *An. arabiensis* may be explained by the lower selection pressure on the indoor feeding *An. gambiae* s.s. due to a lack of indoor vector control tools available in the area [33, 34]. A Knowledge, Attitudes and Practice survey (KAP) performed by MSF in 2013 showed that Shamwana, Baraka and Kashuga had poor coverage and usage of LLINs (unpublished data).

*Anopheles gambiae* s.l. tested by ELISA showed a *P. falciparum* sporozoite rate of 1.0, 2.1 and 13.9% in Shamwana, Baraka and Kashuga respectively. *Anopheles funestus* had a positivity rate of 1.8% in Shamwana and 4.4% in Baraka. In Kashuga no *An. funestus* were collected. Except for the high sporozoite positive rate found in Kashuga the overall sporozoite rates are in line with the findings of the President’s Malaria Initiative (PMI) in 2015–2016. They found a mean sporozoite rate of 5.1% in *An. gambiae* s.l. and 3.3% in *An. funestus* s.l. in seven sentinel sites in different provinces in DRC [37]. Malaria transmission was highly heterogeneous in the study area. Especially in Baraka, a town with a total surface area of approximately 10 km^2^, and Kashuga, a town of approximately 2 km^2^, the mosquitoes carrying *P. falciparum* sporozoites were concentrated in one specific area of approximately 250 × 250 m (Fig. 2). This is critical information for the implementation of vector control interventions. The high EIRs shows that the entire area is in need of effective vector control interventions, however, uniformly applied vector control interventions will be very challenging in this context. Therefore, targeting these specific high transmission spots will be a good start. However, there is no conclusive evidence that targeting these specific high transmission sites will actually reduce the transmission in the entire area [38].

What is driving these spatial transmission foci was not further investigated. However, the presence of fishponds and agricultural fields in Ibuga camp (Kashuga) probably contributed to a higher number of mosquitoes present and in combination with a non-immune population that has been displaced to a malaria endemic area, this might have contributed to the high transmission intensity of malaria in this particular area. The presence of brick production sites in Mushimbakye (South Kivu) might have contributed to the higher number of mosquitoes present. It should be noted that the EIR calculated in this study should be treated with caution, since the collection of mosquitoes took place in a specific short time period and, therefore, the numbers were extrapolated to calculate the monthly EIR. Furthermore, CDC light trap collections were used instead of human landing catches.

In all three sites, the mosquitoes showed resistance to pyrethroids. Clear resistance towards permethrin was observed in all three locations. This is in line with the study of Wat’senga et al*.* for which they performed pyrethroid resistance intensity tests in 11 provinces in DRC and confirmed pyrethroid resistance, with in general higher resistance levels to permethrin than to deltamethrin [39]. It is debatable whether this resistance can be explained by the presence of *kdr* mutations. Both L1014F and L1014S *kdr* mutations were present in this study. Overall, more than 90% of the mosquitoes had at least one of these mutations. However, because of the use of two separate assays in this study to type the different *kdr* mutations, many mosquito samples showed unresolved allele combinations, in which more than two alleles were detected. Therefore, these results were not discussed but have been included in Additional File 1. Mixture of the two alleles is increasingly common especially in central Africa including DRC, however these mixtures are the most problematic for the traditional detection methodologies [15, 16]. This study underscores the need to type both *kdr* mutations via a single detection method like described in Lynd et al*.* [15]. Also, some studies have shown the presence of metabolic resistance, as well as an increase in the cuticle thickness in mosquitoes in other parts of DRC [15, 16, 40, 41]. These resistance mechanisms might also play a role in the mosquitoes collected during this study, but this was not further evaluated. The resistance towards the pyrethroid α-cypermethrin may be explained by the type of LLINs deployed and by the IRS product used in the three areas. To our knowledge, these were the first insecticide susceptibility tests with α-cypermethrin performed in DRC at that time. Resistance towards pyrethroids might also be the result of the use of the same class of insecticides in agriculture [42, 43]. Bendiocarb, pirimiphos-methyl and malathion caused high mortalities and can therefore be considered as candidate insecticides for vector control activities. However, to prevent rapid development of resistance towards these insecticide classes, a clear rotation strategy of different insecticide families is recommended.

Special attention is needed for IDPs. The living conditions of IDPs are generally very poor and because the majority of them moves from one place to another frequently, it is a challenge to protect them with conventional vector control methods such as LLINs and IRS. Options for vector control methods that focus more on the community level rather than the household level, such as environmental management, (biological) larviciding, push–pull systems and spatial repellents, should be further investigated so they can be used in IDP camp settings as a supplement or alternative to LLINs and IRS [44–46].

## Conclusions

At the start of this study in 2014, this was one of the first studies to report insecticide resistance in local malaria vector mosquitoes in the eastern part of DRC. Both *An. gambiae* and *An. funestus* showed high levels of insecticide resistance towards pyrethroids. This study strongly suggests that the risk of malaria in this part of DRC is high, and that malaria prevention methods used in the population are only partially effective with high exposure to (infected) mosquitoes persisting. The results triggered a careful review of the existing vector control strategy in the areas. This, in turn, resulted in the selection of new LLINs with the synergist PBO, and in the selection of new insecticides for IRS with the active ingredients bendiocarb and pirimiphos-methyl. Furthermore, it caused the reinforcement of correct usage and maintenance of LLINs, to maximize not only the mass effect of LLINs, but also the individual protection. The application of larvicides in selected mosquito breeding sites in Baraka and Kashuga has also been added recently to the vector control interventions as a result of this study. The impact of these interventions will remain subject of further study.

## Supplementary information

**Additional file 1**: Excel sheet with details of *kdr* genotypes from mosquito samples collected via CDC light traps and Excel sheet with details on *kdr* genotypes from mosquitoes exposed to different insecticides in the WHO insecticide susceptibility bioassay.

## Data Availability

Data are available on request in accordance with MSF’s data sharing policy at data.sharing@msf.org.

## Abbreviations

CDC: Center for Disease Control, USA
DDT: Dichloro-diphenyl-trichloroethane
DRC: Democratic Republic of the Congo
EIR: Entomological inoculation rate
ELISA: Enzyme-linked immuno sorbent assay
IDP: Internally displaced people
IRS: Indoor residual spraying
*Kdr*: Knockdown resistance
LLIN: Long-lasting insecticide-treated net
MSF: Médecins Sans Frontières
OCA: Operational Centre Amsterdam
PCR: Polymerase chain reaction
UNHCR: United Nations High Commissioner for Refugees
*Vgsc*: Voltage gated sodium channel
WHO: World Health Organization
WHOPES: World Health Organization Pesticide Evaluation Scheme
